# A framework for selecting and assessing soil quality indicators for sustainable soil management in waste dumps

**DOI:** 10.1038/s41598-024-58930-x

**Published:** 2024-04-11

**Authors:** Yue Li, Hongbao Zhao, Jiashun Liu, Chen chaonan, Guo Yuxuan

**Affiliations:** 1 School of Energy and Mining Engineering, China University of Mining and Technology-Beijing, Beijing, 100083 China; 2 College of Civil Engineering, Liaoning Technical University, Fuxin, 123032 Liaoning China

**Keywords:** Open-pit coal mine, Waste dump, Reclamation, Soil quality, Minimum data set, Nemerow index, Ecology, Environmental sciences, Environmental social sciences, Energy science and technology

## Abstract

The primary objective of this study was to develop soil quality indexes (SQIs) to reveal the changes in SQ during the restoration of vegetation in the reclaimed waste dumps of the Hequ open-pit coal mine. The study built an SQI evaluation model for waste dumps based on the soil management assessment framework. The total data set (TDS) consisted of nine physicochemical property indicators. The selection of the minimum data set (MDS) involved the utilization of principal component analysis (PCA) and Norm values. The SQ was comprehensively evaluated for nine indicators, taking into account the non-linear membership function and the improved Nemerow index. The findings suggested a notable disparity in the SQ between the reclaimed area and the unreclaimed area, yet the overall SQ fell short. In the TDS index system, the organic matter has the highest weight and a greater contribution to the soil quality of the waste dumps. In the MDS indicator system, the weights of organic matter and total nitrogen are both 0.5. According to Nemerow index method, the average SQIN of 5 plots is calculated to be 0.4352 ± 0.194. The average value obtained from TDS is 0.581 ± 0.236, and the average value obtained from MDS is 0.602 ± 0.351. The weighted additive method was employed to compute three SQIs, all of which yielded satisfactory outcomes. And the above evaluation methods indicate that the overall soil quality level of the waste dumps is at a moderate level. The sequence of SQ in various waste dumps was as follows: No.4_lower_ > No.1 > No.2 > No.3 > No.4_upper_. Specifically, the non-linear membership function indicated that pH, available nitrogen (AN), available phosphorus (AP), surface moisture content (SMC), and bulk density (BD) were crucial in limiting SQIs in total waste dumps. The crucial limiting SQIs in unreclaimed areas were total phosphorus (TP) and total nitrogen (TN). This analysis demonstrates its efficacy in formulating strategies for the SQ evaluation and targeted soil reclamation plans of waste dumps.

## Introduction

Economic development has been greatly influenced by the extensive exploitation of mineral resources, the destruction of a large area of land caused a range of ecological and environmental issues, including soil erosion and slope instability, etc. It even gave rise to the deterioration of the environment in mining regions and hindered the progress of sustainable development^1–3^. The waste dump is where the rock mass and certain waste materials are stripped off during the manufacturing procedure of an open-pit coal mine. The main components of it consist of the stripped topsoil and rock obtained through open-pit mining. Consequently, the soil of open-pit coal mine waste dumps often suffers from inadequate capacity for soil and water conservation^4^. The lack of greening reclamation often leads to poor reclamation effects and low survival rates for plants. It is almost impossible to restore soil fertility without the ecosystem's self-restoration ability.

It is imperative to comprehend the influence of reclamation on soil quality (SQ) and function to implement effective soil restoration strategies for a particular area. SQ is the ability of the soil to support and maintain the growth of crops. The importance of soil to humans cannot be understated, as it not only boosts production but also improves the ecological environment of mining areas. Hence, the primary objective of sustainable land utilization and administration is to enhance SQ. Doran, JW et al.^5^ suggested measuring SQ in various soil management evaluations. Subsequently, they issued early indicators to identify the factors that restrict SQ in problematic areas. Previous studies on SQ have largely concentrated on assessing SQ, and domestic and foreign scholars have conducted investigations on soil quality index models. SQI is speculated to be a successful method for measuring the impact of various waste dumps on SQ. This tool can integrate valuable information for decision-making^6^. There are different types of methods for establishing SQI, such as the Expert Opinion Index (SQIEOI), which uses the experience and knowledge of trained personnel to select the indicators to be included and determine their importance; the Additive Index (SQIA), an analysis index that considers them to exhibit linear behavior^7,8^; Weighted additive index (SQIW), using weights previously established by experts in the indicators to be included^7,8^; Nemoro Index (SQIN), used for soil quality research around the world^7,8^; And establish a unified weighted additive index (SQIU) based on statistical techniques for indicator weights. Cherubin, MR et al.^9^ chose six indicators based on soil in Brazil as the research object. The soil management assessment framework (SMAF) was utilized to identify the primary soil limiting factors, followed by the prioritization of specific management actions. A scoring function was used by NAKAJIMA, T et al.^10^ to analyze and evaluate the soil quality index (SQI) for different agricultural management practices. Some scholars have also used systematic analysis methods to study soil quality evaluation to improve SQ. Various techniques, including principal component analysis (PCA)^11^ and the minimum data set (MDS)^12,13^ are employed to eliminate overlapping information on indicators. Li Guilin et al.^14^ investigated the technique for ascertaining the MDS for soil quality evaluation. However, the SQIW method can select indicators unrelated to the problem or phenomenon being studied, as it is based on previously established indicators. In contrast, the SQIN method is not influenced by human subjectivity, so it can mathematically select the indicators most closely related to the problem or phenomenon of interest. This can ensure a more accurate selection of relevant indicators.

The restoration of soil fertility is the core content and ultimate goal of ecosystem restoration in the open pit coal mine waste dumps. However, there are few reports on checking soil fertility indicators and assessing the SQ of the waste dumps. Therefore, this study focused on various waste dumps of the Hequ open-pit coal mine and established two evaluation models, SQIW and SQIN, with the same indicator set. The soil fertility indicators of five plots were measured through on-site investigation. The improved Nemerow index and the MDS method were used to evaluate the SQ of each waste dump in the Hequ open-pit coal mine. The key limiting factors affecting the reclamation area were determined in the same indicators, and a more feasible unified evaluation model was explored for soil improvement, ecological restoration and reconstruction, and ecological environment security in similar mining areas.

## Materials and methods

### Study area

**Figure 1:**
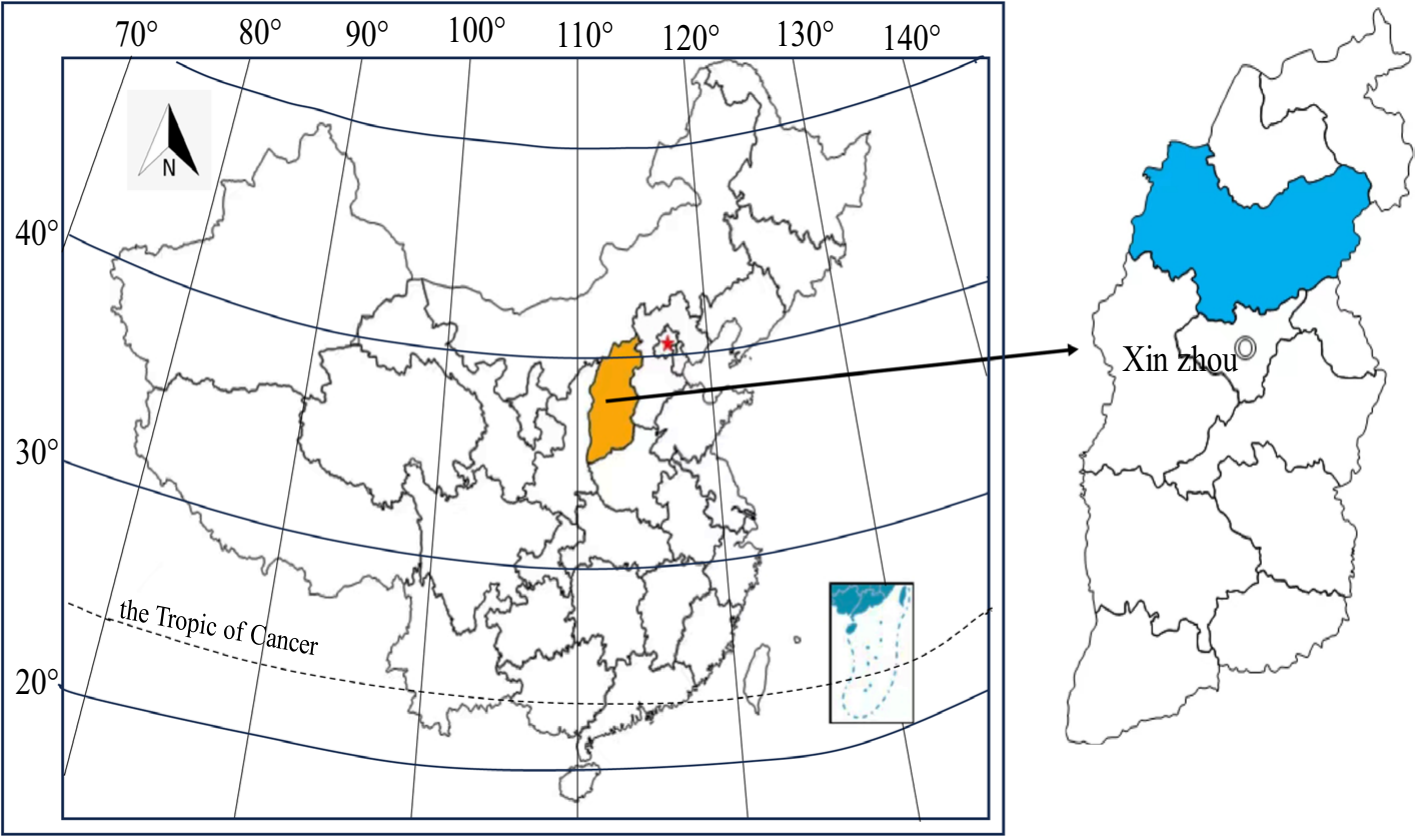
Geographic location.

**Figure 2:**
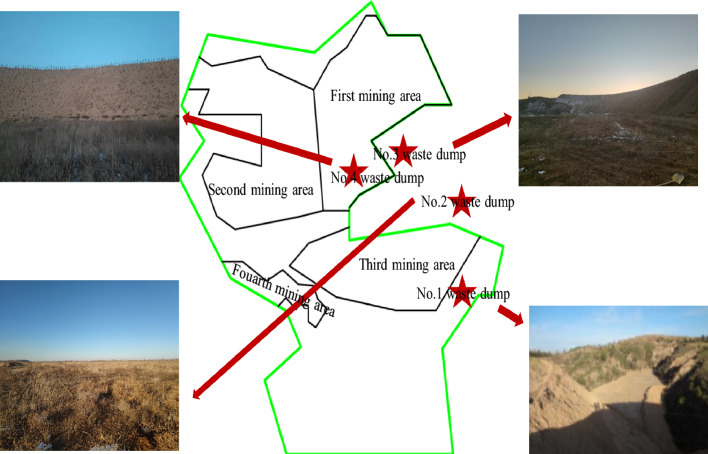
Current situation of the study area.

The study was conducted at the the Hequ open-pit coal mine dump (Fig. 1) situated on the Loess Plateau. The first mining area (Fig. 2) had now been mined to No.13 Coal Seam. The depth extended between 870 m above sea level and 1060 m. The stripping of the No.13 coal seam and its overlying layers in the first mining area has led to an augmentation in the mining depth, resulting in the gradual complete coverage of the No.4 waste dump. The mining area has executed pertinent governance initiatives in compliance with the stipulations outlined in the "Green Mine Construction Plan for Shanxi Coal Import and Export Group Hequ Jiuxian Opencast Coal Industry Co., Ltd." furnished by the Hequ open-pit coal mine. The untreated sections of the No.1, No.2, No.3, and No.4 waste dumps respectively, measured 82.21 hm^2^ (73.88 hm^2^ after deducting reclaimed land), 108.99 hm^2^ (95.56 hm^2^ after deducting reclaimed land), 32.99 hm^2^, and 42.16 hm^2^. The No.1 and No.2 waste dumps have implemented some control measures as required, and have undergone reclamation and greening. According to the upstream water collection capacity of the external waste dumps, one masonry retaining wall was designed to be installed at the downstream ditch mouth of the No.1 and No.2 waste dumps respectively. The platform was filled and formed for the No.4_lower_ waste dump. Planting bags were set up in the No.3 and No.4_lower_ waste dump. The slope was naturally restored to grassland, and the vegetation type was mostly Artemisia. The urgent issues to be addressed in the greening and reclamation of the outer waste dumps of the Hequ open-pit coal mine are poor maintenance of green plants, poor growth, and unsatisfactory greening effects, etc. Greening and reclamation measures have not yet been taken for the No.3 and No.4_lower_ waste dumps. The drainage engineering of the four outer waste dumps was relatively rudimentary, and no drainage ditches have been constructed. Under the influences of rainfall and other weather conditions, it was extremely easy to cause disasters such as landslides, mudslides, etc., and even pose significant safety hazards. Additionally, the supporting roads for the reclamation of the waste dumps have not been constructed.

### Collection and analysis of samples

The No.1–4 four waste dumps in the mining area underwent sample collection on the reclaimed land with varying levels of reclamation and fertility conditions. The waste dumps were categorized into the reclaimed area at No 1, 2 and the the lower reclaimed area of No.4 waste dumps (hereinafter denoted as 4_lower_), as well as the unreclaimed area at the No.3 waste dump and the upper unreclaimed area of No. 4 waste dumps (hereinafter denoted as 4_upper_). The unregulated section of the No.1 and No.2 waste dumps served as a site for waste disposal, where waste materials were mixed with the No.4_upper_ and No.3 waste dumps simultaneously. Consequently, the soil quality level was comparable. Each sample plot set consisted of an equal distribution of 5 sampling points. For each sampling point, five identical points were tablelished by the "X" shape. The soil drill method was used to conduct multi-point mixed sampling on the 0–30 cm soil layer after removing the humus and roots of weeds and vegetation on the surface layer. The study mixed soil samples from the same sampling point and depth into one soil sample. Sterile preservation bags were used to place the soil samples, which were then transported back to the laboratory. Following the process of air drying, they underwent a 2 mm sieve to conduct further soil analyses. A high-precision^15^ soil pH tester was used to measure the soil's pH. The flow analyzer was used to ascertain the quantities of total nitrogen and total phosphorus. The ring knife^16,17^ method yielded bulk density. The YT-TRA soil nutrient detector was utilized to ascertain the quantity of accessible nutrients.

## Soil quality evaluation of waste dumps

### Selection of evaluation index system and membership function

The study took into account the significant attributes of soil and water loss in the waste dump of the Hequ open-pit mine in the Yellow River Basin^18^ and consulted the standardized Tablele for soil nutrient content in China^19^ (Table 1) and pertinent research results^6,20–27^. Hence, nine indicators were chosen for the waste dumps, encompassing soil physical and chemical properties that assess the soil's ability to retain water and fertilizer, including soil organic matter (SOM), available nitrogen (AN), available phosphorus (AP), available potassium (AK), pH, total phosphorus (TP), total nitrogen (TN), surface moisture content (SMC) and bulk density (BD). Nine indicators were used to evaluate the impact of reclamation on the SQ of the waste dumps during the membership process. Soil organic matter, total nitrogen, total phosphorus, available phosphorus, and available potassium were all classified as S-type membership functions. The definitions of bulk density, pH, and surface moisture content are used as parabolic membership functions, taking into account the existence of appropriate critical ranges. The turning points of the S-type membership functions were determined by selecting the minimum value m_1_ and maximum value m_2_ from the measured values of each indicator as the turning points of the function. The point at which the index of the parabolic membership function reached its turning point was determined by analyzing both literature and field measurement data. The tableulated data displayed the membership function and parameters of the evaluation indicators for SQ in the waste dumps (Table 2).

**Table 1:** Unified standards for soil nutrient content in China^19^.

Nutrient indicators	6	5	4	3	2	1
pH	< 4.5	4.5–5.5	5.5–6.5	6.5–7.5	7.5–8.5	> 8.5
Soil organic matter (g·kg^−1^)	< 6	6–10	10–20	20–30	30–40	> 40
Available nitrogen (mg·kg^−1^)	< 30	30–60	60–90	90–120	120–150	> 150
Available phosphorus (mg·kg^−1^)	< 3.0	3.00–5.00	5.00–10.00	10.00–20.00	20.00–40.00	> 40.00
Available potassium (mg·kg^−1^)	< 30	30.00–50.00	50.00–100.00	100.00–150.00	150.00–200.00	> 200.00
Total phosphorus (g·kg^−1^)	< 0.5	0.5–0.75	0.75–1.0	1.1–1.5	1.5–2.0	> 2.0
Total nitrogen (g·kg^−1^)	< 0.5	0.5–0.75	0.75–1.0	1.1–1.5	1.5–2.0	> 2.0

**Table 2:** Membership function and parameter of evaluation indicators for Waste Dump soil quality.

Index	Effect of soil attribute index on crop yield	Membership function	Membership function parameter			
			m_1_	m_2_		
Soil organic matter (g·kg^−1^)	+	$$f(x) = \left\{ {\begin{array}{*{20}l} {1.0,} \hfill & {m \ge m_{2} } \hfill \\ {\frac{{0.9\left( {m - m_{1}} \right)}}{m_{2} - m_{1}} + 0.1,} \hfill & {m_{1} \le m < m_{2} } \hfill \\ {0.1,} \hfill & {m < m_{1} } \hfill \\ \end{array} } \right.$$	4	13		
Available nitrogen (mg·kg^−1^)	+		5	15		
Available phosphorus (mg·kg^−1^)	+		3	10		
Available potassium (mg·kg^−1^)			100	380		
Total phosphorus (g·kg^−1^)	+		1	2		
Total nitrogen (g·kg^−1^)	+		1	2.5		

			x_1_	x_2_	x_3_	x_4_
Surface moisture content (%)	+ −	$$f(x) = \left\{ {\begin{array}{*{20}l} {\frac{{0.9\left( {x_{4} - x} \right)}}{{x_{4} - x_{3} }} + 0.1,} \hfill & {x_{3} \le x < x_{4} } \hfill \\ {1.0,} \hfill & {x_{2} < x < x_{3} } \hfill \\ {\frac{{0.9\left( {x - x_{1} } \right)}}{{x_{2} - x_{1} }} + 0.1,} \hfill & {x_{1} \le x < x_{2} } \hfill \\ {0.1,} \hfill & {x < x_{1} } \hfill \\ \end{array} } \right.$$	5.25	8	10	16
Bulk density (g·kg^−1^)	+ −		0.55	0.75	0.85	1.25
pH	+ −		5.5	6.5	7.5	8.5

“ + ” indicates that the relationship between soil index and yield is S-shaped curve; “−” indicates an inverse S-shaped curve relationship between soil indexes and yield; “ + −” indicates a parabolic curve relationship between soil indexes and yield; x_1_, x_2_, x_3_ and x_4_ are the critical values of membership function respectively. The same below.

### Construction of minimum data set

A principal component analysis (PCA) and Norm values were performed on the standardized data matrix of the TDS to avoid overlapping information between the primary indicators and reduce the number of participating indicators representing the MDS. The study found that the Pearson correlation coefficient was the best way to determine the choice of indicators. If the correlation coefficient between indicators in a group was less than 0.5, all indicators would be kept. If there is a strong correlation (r > 0.5) between indicators, the indicator with the highest, norm value will be chosen to enter be included in the MDS. To construct the MDS, through principal component analysis combined with Norm values were as follows: a PCA was performed on the standardized data matrix of the TDS. The principal components (PCs) eigenvalues greater than or equal to 1 and those that accounted for a minimum of 5% of the overall variation in the dataset were selected to identify MDS. Indicators exhibiting a load equal to or greater than 0.6 on the identical PC were consolidated into a unified group. If an indicator exhibited a load of 0.6 or higher on two PCs concurrently, it would be amalgamated into a cluster exhibiting diminished correlation with other indicators^25^; Conversely, if an indicator displayed a load of 0.6 or lower, it would be partitioned into the group exhibiting the highest load. In addition to other indicators. For each PC, only indicators with a loading value within 10% of the highest weighted loading were kept as significant indicators for indexing this PC. Pearson's correlation analysis (Table 6) was employed to ascertain indicator redundancy when more than one indicator was retained in each PC. If there was no correlation between the highly loaded indicators, each indicator was kept in the MDS. Otherwise, only the indicator with the greatest weighted loading was chosen for the MDS^28^. As the Norm value of the indicator, so does its capacity to interpret comprehensive information. The evaluation indicators were evaluated using the Norm value as follows

1 $$N_{ik} = \sqrt {\sum\limits_{i = 1}^{k} {\left( {U_{ik}^{2} \lambda_{k} } \right)} }$$

where $N_{ik}$ is the comprehensive load of the $i$ variable on the kPC with eigenvalues ≥ 1, $U_{ik}$ is the load of the $i$ variable on the PC, and $\lambda_{k}$ is the eigenvalue of the kPC.

### Weights of evaluation indicators

The PCA-derived common factor variance may indicate the contribution of a specific indicator on the overall variance. The larger the common factor variance, the greater its contribution to the overall variance^29^. The weight value of each indicator was calculated using PCA in this study. The soil indicator’s weighting values of the common factor variance of each indicator by the total common factor variance of all indicators^28^.

### Establishment of soil quality index for waste dumps

(1)(1) After obtaining the weights and membership degrees of the indicators mentioned above, a model for calculating the soil quality index of waste dumps (WDSQI) based on the weighted additive methods was the soil quality index (SQI) model. The modified indicator scores were assimilated into a soil Quality Index of waste dumps (WDSQI) through the utilization of weighted additives. The following are the methods (Eq. (2)) to be employed:
  2 $$WDSQI = \sum\limits_{i = 1}^{n} {W_{i} S_{i} }$$

where $WDSQI$ is The soil quality index of waste dumps, $W_{{_{i} }}$ is the Weights of the i variable, $S_{{_{i} }}$ is the membership degree of the $i$ variable, $n$ is the number of SQ evaluation indicators for waste dumps.

$TDS - WDSQI$(representing the measured value) is the soil quality index of the waste dumps calculated from TDS, $MDS - WDSQI$ (representing simulated values) is the soil quality index of the waste dumps obtained from the MDS^30,31^.

(2)(2) The Nemerow index^32,33^ was frequently employed to assess the levels of soil pollution and water quality. It has the potential to be enhanced as a means of evaluating the quality of soil nutrients and fertility. The Nemerow index was a weighted multi-factor environmental quality index. It accounted for extreme or prominent maximum values, considering various environmental quality factors. Particularly, the factors that had the most significant influence were taken into account. The Nemerow environmental quality index circumvented the impact of subjective factors during the weighting process and was presently an extensively employed environmental quality index. The barrel theory posits that the nutrient index exhibits the highest level of deficiency. The fundamental equation was as follows:
  3 $$P_{N} = \sqrt {\overline{P}_{i} + P_{i\max }^{2} /2}$$

where $P_{N}$ is soil quality index, $\overline{P}_{i}$ is the average value of the quality index of $i$ variable in the sample,$P_{i\max }$ is the maximum value of the quality index of $i$ variable for various samples.

Combining the Nemerow index with both extreme values, the Nemerow index method was improved by replacing $P_{i\max }$ in the original formula with $P_{i\min }$, thereby reflecting the soil level’s limiting factors through the analysis of the minimum index value. The improved Nemerow index emphasized the impacts of the most unfavorable indicators of soil properties on soil fertility, mirroring the minimum factor law of crop growth. The improved Nemerow index was as follows:

4 $$P_{N} = \sqrt {\frac{{\overline{P}_{i}^{2} + P_{i\min }^{2} }}{2}} \times \frac{n - 1}{n}$$

where $P_{i\min }$ is the minimum value of the quality index of $i$ variable for various samples, $n$ is the number of participating evaluation factors.

The addition of correction terms was to improve the credibility of the evaluation results. The more soil quality evaluations there are, the more credible the results will be. The Nemero index was further modified by merging the membership function. The improved Nemerow index was as follows:

5 $$WDSQIN = \sqrt {\frac{{\mathop {\overline{f}_{i} }\nolimits^{2} + \mathop {f_{i\min } }\nolimits^{2} }}{2}} \times \frac{n - 1}{n}$$

where $\overline{f}_{i}$ is the average value of the membership degree of factor nutrient of each i variable, $F_{i\min }$ is the smallest among the membership degrees of each single factor nutrient.

The utilization of the deformed Nemerow index in the computation of the all-encompassing fertility index of soil could accurately depict the effects of the index characterized by the smallest degree of nutrient membership (the most deficient content). The IFI and membership values fall within the interval of [0.1, 1.0]. Likewise, as the IFI value approached 1, the soil fertility increased.

(3)(3) The WDSQI was evenly distributed into five levels. Detailed classification levels and standards are shown in Table 3.

**Table 3:** Classification Standards for Soil Quality Assessment of Waste Dumps.

Fertility degree	Excellent	Good	Medium	Deficient	Destitute
WDSQI	> 0.8	0.7–0.8	0.6–0.7	0.4–0.6	< 0.4

### Data processing

The SPSS 26.0, Origin 2018, and Excel statistical software packages for Windows were utilized for all statistical analyses. The data underwent PCA and bivariate correlation analysis using SPSS26.0, while Origin 2018 was employed for linear fitting. The coefficient of variance (CV) was employed to illustrate the dispersion and difference of each SQI.

## Results and analysis

### Statistics of soil quality evaluation indicators for waste dumps

Generally, the physical and chemical properties of soil are examined to reflect the level of soil quality in waste dumps. The soil properties of the waste dumps vary significantly, and the data regarding the assessment of SQ for each sample site is presented in Table 4. The pH of the unreclaimed area was significantly higher than that of the already reclaimed area, with the 4_upper_ having the most significant natural recovery and the No.4_lower_ having the least. The No.4_upper_ and No.4_lower_ had values of 8.886 and 8.266, respectively. The content of SOC, AN, AP, AK, TP, and TP in the natural recovery area was significantly lower than that in the reclaimed area (*P* < 0.05). The No. 3 and No.4_upper_ had a SOC content of 5.160 g/kg and 4.120 g/kg, respectively. The concentrations of No. 1, No. 2, and No.4_lower_ were 12.160 g/kg, 11.640 g/kg, and 12.280 g/kg respectively. The No.1 contained the highest concentration of alkaline nitrogen, measuring 12.908 mg/kg. The No.4_upper_ contained a minimum of 5.392 mg/kg. The No.4_lower_ exhibited the highest levels of AP, TN, TP, measuring 9.13 g / kg, 2. 766 g/kg, and 2.308 g/kg, respectively. The No.4_upper_ had the lowest values of 4.884 g/kg, 0.966 g/kg, and 0.804 g/kg, respectively. The No.1 exhibited the AP, reaching 399.00 mg/kg, while the No.4_upper_ had the lowest content of 259.00 mg/kg. There was no significant difference in BD and SMC among different waste dumps (*P* > 0.05).

**Table 4:** Statistics of soil quality evaluation index in different waste dumps.

Index	The No.1	The No.2	The No.3	The No.4_upper_	The No.4_lower_
pH	8.294 ± 0.294bc	8.494 ± 0.190c	8.80 ± 0.470ad	8.89 ± 0.153ad	8,267 ± 0.117cd
SOM/(g·kg-1)	12.160 ± 2.69bc	11.640 ± 1.50bc	5.160 ± 1.1415abd	4.12 ± 0.356abd	12.280 ± 1.77cd
AN/(mg·kg-1)	13.69 ± 3.317bc	12.622 ± 2.440bc	6.078 ± 0.689abd	5.392 ± 1.559abd	11.348 ± 2.560cd
AP/(mg·kg-1)	8.326 ± 1.225c	7.778 ± 2.168	5.544 ± 1.234d	4.884 ± 0.876ad	9.130 ± 2.173cd
AK/(mg·kg-1)	399.00 ± 35.164bc	388.40 ± 26.17bc	303.60 ± 43.28abd	259.00 ± 34.45abd	397.60 ± 18.12cd
TP/(g·kg-1)	1.870 ± 0.42bc	1.746 ± 0.41c	0.954 ± 0.44ad	0.804 ± 0.48abd	2.308 ± 0.42cd
TN/(g·kg-1)	2.455 ± 0.346bc	1.816 ± 0.485d	0.982 ± 0.531ad	0.966 ± 0.582ad	2.766 ± 0.2115bcd
SMC/(%)	9.868 ± 3.021	8.534 ± 3.247	6.178 ± 2.852	6.356 ± 2.704	10.282 ± 3.296
BD/(g·kg-1)	0.778 ± 0.181	0.548 ± 0.285	0.586 ± 0.171	0.594 ± 0.154	0.886 ± 0.156

Different lowercase letters indicate significant differences (*P* < 0.05) among the different sites under the same indicator based on one-way ANOVA followed by LSD test.

*BD* Bulk density; *SOM* Organic matter; *TN* Total nitrogen; *AN* Available nitrogen; *TP* Total phosphorus; *AP* Available phosphorus; *TK* Total potassium; *AK* Available potassium; *SMC* Surface moisture content.

The indicators’ sensitivity is typically indicated by the CV, which was categorized into four categories: Insensitive index CV < 10%; Low sensitivity index 10% ≤ CV < 50%; Moderate sensitivity index 50% ≤ CV < 100%; Strong sensitivity index CV ≥ 100%. Nine soil indicators of SQ were evaluated as potential indicators associated with various waste dumps. The CV of PH was 4.04%, indicating a lack of sensitivity. The coefficients of variation for SOM, TN, TP, AN, AK, AP, SMC, and BD exhibited low sensitivity, with percentages of 44.29%, 25.82%, 29.36%, 40.89%, 33.34%, 18.84%, 39.73%, and 32.86%, respectively. The soil capacity index of the dump exhibited significant variability.

The same below.

### The minimum data set

The PCA analysis revealed that the two PCs had eigenvalues greater than 1.0 and accounted for more than 73.769% of the variance of the original data (Table 5). The first two PCs demonstrated a high level of explanatory power. The PC1 accounted for 60.39% of the overall variance. The loading value of SOM was the highest, with only pH falling within the 10% range of the highest loading value. There was a significant correlation (*P* < 0.01) between these two indicators (Table 6). It was evident that the loading value for the two principal components varies significantly between PC1 and PC2. The variations in the explanatory capacity of various principal component indicators on overall variance were also evident. The eight primary indicators such as pH SOM, AN, AP, AK, TP, TN, and SMC in PC1 had an absolute loading value of more than 0.6. The data indicated that these indicators have a high contribution rate in PC1. In PC2, BD and pH were selected as the primary indicators, and the absolute loading value was greater than 0.6. The highest contribution rate of pH and BD was observed in PC2, as indicated.

**Table 5:** Load matrix and norm values for each index.

Index	PC1	PC2	Group	Norm
SOM	0.899	− 0.325	1	4.520
AN	0.801	− 0.412	1	3.691
TN	0.795	0.386	1	3.614
AP	0.780	0.098	1	3.318
AK	0.779	− 0.476	1	3.571
SMC	0.719	0.284	1	2.907
TP	0.784	0.111	1	3.355
pH	− 0.815	0.87	2	4.521
BD	0.585	0.665	2	2.392
Eigenvalue	5.435	1.204		
Variance /%	60.392	13.376		
Cumulative variance /%/	60.392	73.769

**Table 6:** Correlation coefficient matrix of primary selection indicators for soil quality evaluation of waste dumps.

	pH	SOM	AP	AN	AK	BD	SMC	TN	TP
pH	1								
SOM	− 0.386**								
AN	− 0.535**	0.735**	1						
AP	− 0.603**	0.841**	0.542**	1					
AK	− 0.645**	0.832**	0.566**	0.763**	1				
BD	− 0.761**	0.330	0.518**	0.262	0.299	1			
SMC	− 0.500*	0.589**	0.652**	0.457*	0.472*	0.522**	1		
TP	− 0.735**	0.633**	0.497*	0.533**	0.574**	0.533**	0.546**	1	
TN	− 0.532**	0.658**	0.628**	0.487*	0.487*	0.679**	0.658**	0.689**	1

** indicates a significant difference (*P* < 0.01), * indicates a significant difference (*P* < 0.05).

In both PC1 and PC2, pH achieved a load of at least 0.6. The correlation coefficient between pH and the PC1 of indicators, ranging from 0.386 to 0.751, and the correlation coefficient with PC2 of indicators, which was 0.761, resulted in the smallest overall correlation with the PC1 of indicators (Table 6). Consequently, pH was selected as the indicator of PC1. Compare the normal values of each group using the MDS principle, and select indicators with normal values within 10% of the highest load value in each group. The initial indicators chosen encompassed SOM, PH, and BD. The correlation coefficient between SOM and pH was less than 0.5, as determined by the Norm value and correlation analysis. As a result, PH and SOM were selected to be part of the MDS. The PC2 contained solely BD. As a result, BD was elected to be a part of the MDS. The MDS contained three indicators: SOM, pH, and BD. The percentage of individuals being screened amounted to 77.8%. The soil quality evaluation system for waste dumps was significantly streamlined, reducing the influence of overlapping information among evaluation indicators.

### Analysis of contribution rate of soil quality indicators

The study used PCA to find the common factor variance between TDS and MDS. The weighting values of TDS and MDS (Table 7) were further computed. Originating from Table 7, It was evident that the soil fertilizer retention capacity and soil water retention index had respective weighting values of 0.788 and 0.212. The soil fertilizer retention capacity attribute index held the greatest weight. Hence, the capacity to retain soil fertility retention capacity had the greatest contribution to the SQ of the waste dumps and played a crucial role in evaluating SQ. Specifically, SOM held the top position in terms of weight. It indicated a significant contribution of SOM to SQ in the waste dumps. The weights of AK, AP, BD, TN, and pH were all relatively high. The weights of these indicators were all greater than or equal to 0.1. The soil's ability to retain water re was considerable. The attribute indicators of soil and water conservation capacity were the source of two indicators that entered MDS. When evaluating SQ for waste dumps, it was essential to take into account the two primary indicators chosen for MDS. The SOM indicators held the top position in terms of weight among the MDS, with a value of 0.399. The SOM made an equally significant contribution to SQ in the waste dump.

**Table 7:** Common factor variance and weights of MDS and TDS for soil quality evaluation of waste dumps.

Index	TDS		MDS	
	Common factor variance	Weight	Common factor variance	Weight
pH	0.672	0.101	0.702	0.339
SOM	0.914	0.138	0.826	0.399
AP	0.811	0.122		
AN	0.618	0.093		
AK	0.833	0.125		
BD	0.785	0.118	0.540	0.261
SMC	0.598	0.090		
TP	0.627	0.094		
TN	0.780	0.118		

### Minimum data set accuracy verification

**Figure 3:**
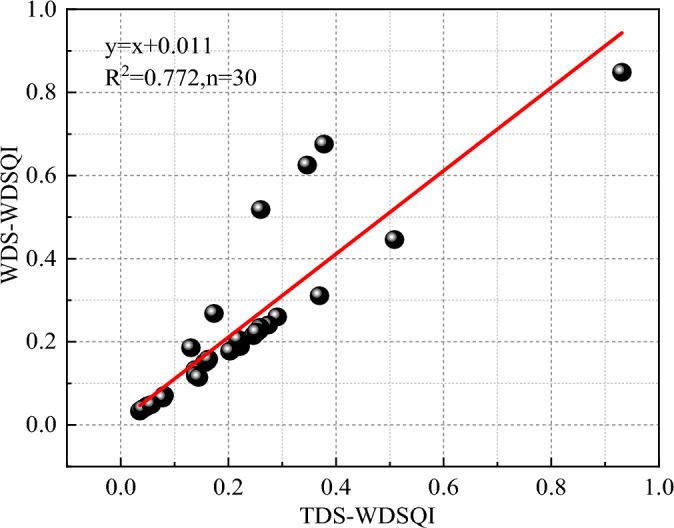
Correlation of dump quality index based on TDS-WDSQI and MDS-WDSQI.

Verifying the rationality of the MDS evaluation index system is an important part of soil quality evaluation. Hence, when utilizing MDS to evaluate the SQ of the waste dumps, it is essential to validate the precision of the evaluation results to guarantee the accuracy of the evaluation. The study utilized data from five waste dumps to validate the evaluation of the MDS. The selection of indicators for MDS had a direct impact on the accuracy of soil quality evaluation in waste dumps. The study applied PCA to each indicator and determined the weight by calculating the common factor variance of each indicator (Table 6). The study standardized each indicator and substituted it into a function to calculate the results of different data sets. The values of the $TDS - WDSQI$ varied between 0.131 and 0.378, with an average of 0.258 ± 0.108. The values of the $MDS - WDSQI$ varied between 0.186 and 0.677, with an average of 0.454 ± 0.217. The variance in average between $TDS - WDSQI$ and $MDS - WDSQI$ was negligible. This study confirmed the accuracy of the MDS indicators through the implementation of regression analysis on $TDS - WDSQI$ and $MDS - WDSQI$. The results depicted in Fig. 3 demonstrated a significant positive correlation between $TDS - WDSQI$ and $MDS - WDSQI$ (R^2^ = 0.772 n = 30). The MDS in this study could more accurately reflect the information of TDS on the soil quality evaluation of each waste dump. And the MDS had good representativeness.

### Analysis of nonlinear membership function

**Figure 4:**
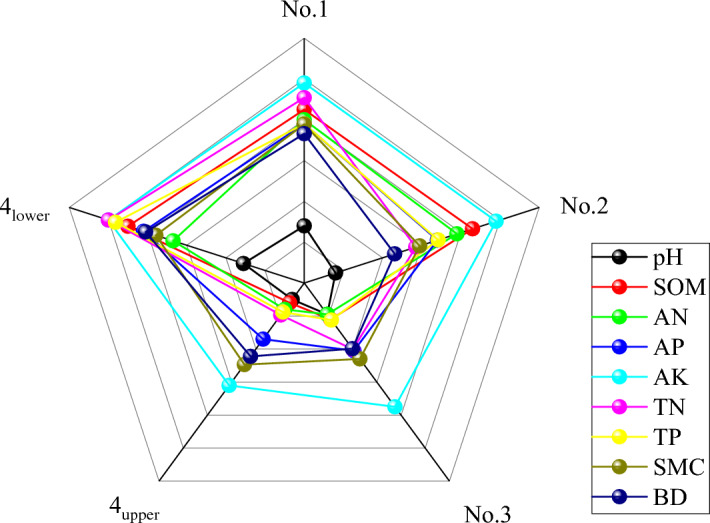
Radar chart of membership function of various soil indexes.

The radar chart was drawn according to the membership value (Table 8). The waste dumps’ soil fertility assessment index (Fig. 4) was analyzed. A radar chart is a useful tool for understanding how a single factor affects soil state and overall fertility quality. And it is suitable for comprehensive analysis of multiple indicators. The radar chart has the potential to directly depict the influences of a single factor fertility index on the condition of the soil and the overall quality of fertility. It is appropriate for a thorough examination of multiple indicators. The greater the degree of membership, the higher the fertility level which is indicative of a single indicator. Originating from Table 1 and Fig. 4, it could be seen that:

**Table 8:** Membership of soil fertility assessment index of waste dumps.

Waste dump type	Number	Membership								
		pH	SOM	AN	AP	AK	TN	TP	SMC	BD
No.1	F-1–1	0.24	1.00	1.00	1.00	1.00	1.00	1.00	0.27	0.71
	F-1–2	0.37	1.00	1.00	0.86	1.00	1.00	0.98	1.00	0.98
	F-1–3	0.13	0.95	0.72	0.76	1.00	0.90	0.65	0.98	0.10
	F-1–4	0.26	0.61	0.78	0.71	0.92	0.95	0.60	0.64	0.87
	F-1–5	0.42	0.7	0.53	0.59	1.00	0.69	0.67	1.00	1.00
	Mean of membership	0.28	0.85	0.8	0.78	0.98	0.91	0.78	0.78	0.73
	Mean of measured values	8.29	12.16	13.67	8.33	399	0.78	9.87	1.87	2.46
No.2	S-2–1	0.16	0.97	0.94	1.00	0.92	1.00	1.00	0.43	0.96
	S-2–2	0.11	1.00	1.00	0.40	1.00	0.54	0.78	1.00	0.10
	S-2–3	0.200	0.73	0.86	0.57	1.00	0.45	0.70	0.79	0.15
	S-2–4	0.10	0.68	0.63	0.63	1.00	0.47	0.34	0.39	1.00
	S-2–5	0.24	0.92	0.49	0.84	0.96	0.39	0.60	0.34	0.10
	Mean of membership	0.16	0.86	0.78	0.68	0.98	0.57	0.68	0.59	0.46
	Mean of measured values	8.49	11.64	12.62	7.78	388.4	0.548	8.53	1.75	1.81
No.3	T-3–1	0.10	0.23	0.28	0.50	0.73	0.31	0.22	1.00	0.33
	T-3–2	0.10	0.16	0.20	0.47	0.54	0.10	0.60	0.10	0.15
	T-3–3	0.10	0.39	0.17	0.10	0.92	0.10	0.10	0.10	0.64
	T-3–4	0.54	0.22	0.22	0.37	0.77	0.50	0.10	0.27	1.00
	T-3–5	0.10	0.10	0.11	0.61	0.81	1.00	0.10	1.0	0.10
	Mean of membership	0.19	0.22	0.19	0.41	0.75	0.40	0.22	0.49	0.44
	Mean of measured values	8.8	5.16	6.08	5.54	303.6	0.59	6.18	0.95	0.98
4_upper_	F-4–1	0.10	0.15	0.10	0.4	0.76	0.52	0.10	1.00	0.15
	F-4–2	0.10	0.10	0.20	0.25	0.69	0.10	0.12	0.10	0.46
	F-4–3	0.10	0.14	0.34	0.22	0.60	0.15	0.46	0.10	0.10
	F-4–4	0.10	0.12	0.10	0.33	0.50	0.10	0.10	1.00	1.00
	F-4–5	0.10	0.10	0.10	0.50	0.53	0.10	0.10	0.10	0.28
	Mean of membership	0.10	0.12	0.16	0.34	0.62	0.19	0.18	0.46	0.40
	Mean of measured values	0.89	4.12	5.39	4.88	259	0.59	6.36	0.80	0.97
4_lower_	F-5–1	0.38	0.97	0.83	0.95	1.00	1.00	1.00	0.13	0.39
	F-5–2	0.23	0.88	0.99	0.71	1.00	1.00	0.98	0.73	0.91
	F-5–3	0.18	1.00	0.45	0.61	1.00	1.00	1.00	1.00	1.00
	F-5–4	0.32	1.00	0.47	1.00	1.00	1.00	0.84	0.93	0.75
	F-5–5	0.44	0.65	0.62	0.82	1.00	1.00	1.00	1.00	1.00
	Mean of membership	0.31	0.90	0.67	0.82	1.00	1	0.96	0.76	0.81
	Mean of measured values	8.27	12.28	11.35	9.13	397.6	0.89	10.28	2.31	2.77

The pH’s membership ranking in five sample plots had the lowest average. The pH levels in various waste dumps were ranked as follows: No.4_lower_ (0.31), No.1 (0.28), No.3 (0.19), No.2 (0.16), and No.4_upper_ (0.1). The No. 3 and No. 4_upper_ exhibited a relatively elevated pH level, specifically at the 1st level. The pH of No.1, 2, and 4_lower_ was at the 2nd level. The data suggested that pH is one of the limiting factors affecting SQ in the area.

The SOM’s average membership ranking in five sample plots was as follows: 4_lower_ (0.90), No.2 (0.86), No.1 (0.85), No.3 (0.22), and 4_upper_ (0.12). Both the SOM of No.3 and No.4_upper_ situated at the 6th level, exhibited a relatively low level of quality. The content of SOM on No. 1, No. 2, and 4_upper_ resided on the 4th level, at the nutrient level. The data suggested that SOM was one of the main limiting factors of SQ in the area., and it was corroborated by the measured value.

The AN’s average membership ranking in five sample plots was as follows: No.1 (0.80), No.2 (0.78), 4_lower_ (0.67), No.3 (0.19), and 4_upper_ (0.16). The content of AN in various waste dumps was nutrient, at the 5th level. The data suggested that AN is one of the main limiting factors affecting the quality of SQ in the area.

The AP’s average membership ranking in five sample plots was as follows: 4_lower_ (0.82), No.1 (0.78), No.2 (0.68), No.3 (0.41), and 4_upper_ (0.34). The content of AP on No. 1, No. 2, No. 3, and 4_lower_ was at the 4th level. Nevertheless, No. 3 was in close proximity to the lowest level of 4th level. The AP had a minimum content of 4_upper_, reaching the 5th level. The data suggested that AP is one of the main limiting factors affecting the SQ in the area.

The AP’s average membership ranking in five sample plots was as follows: 4_lower_ (1.0), No.1 (0.98), No.2 (0.98), No.3 (0.75), and 4_upper_ (0.62). The content of AP in various waste dumps was nutrient, at the 1st level. Consequently, the content of AP in different waste dumps of Hequ open-pit coal mine was plentiful. Therefore, he presence of AP did not impose any constraints on the SQ in the area.

The TN’s average membership ranking in five sample plots was as follows: 4_lower_ (1.0), No.1 (0.91), No.2 (0.57), No.3 (0.40), and 4_upper_ (0.19). The content of TN of 4_lower_ was classified as the 1st level. The content of TN of No.1 and 2 was at the 2nd level. The content of TN of the No.3 and 4_upper_ was relatively low, at the 3rd level. The data suggested that TN is one of the main limiting factors for SQ of the No.3 and 4_upper_.

The TP’s average membership ranking in five sample plots was as follows: 4_lower_ (0.96), No.1 (0.78), No.2 (0.78), No.3 (0.68), and 4_upper_ (0.22). The content of TP of No. 1 and 4_lower_ was classified as the 1st level. The content of TP of No. 2 was at the 4th level. The content of TP of No. 3 and 4_upper_ was comparatively low, at the 4th level. The data suggested that TP is one of the main limiting factors for SQ of the No. 3 and 4_upper_.

The SMC’s average membership ranking in five sample plots was as follows: No.1 (0.78), 4_lower_ (0.76), No.2 (0.59), No.3 (0.49), and 4_upper_ (0 46). It can be inferred that the ideal moisture content of loess should be between 19 and 21%, taking into account the typical soil moisture standards. Therefore, the content of SMC in various waste dumps was at a poor level. The data suggested that SMC is one of the limiting factors affecting SQ in the region.

The BD’s average membership ranking in five sample plots was as follows: 4_lower_ (0.81), No.1 (0.73), No.2 (0.46), No.3 (0.44), and 4_upper_ (0.40). Planting crops, particularly corn, resulted in the reclamation of the waste dumps in the research area. Consequently, based on the corn's ability to sustain the BD within the range of 60–80 g/kg, it indicated that the BD of all waste dumps was at a poor level. It drastically curtailed the cultivation of crops. Obviously, the data suggested that BD is one of the limiting factors affecting SQ in the area.

### Characteristics of soil quality changes in reclaimed waste dumps

**Figure 5:**
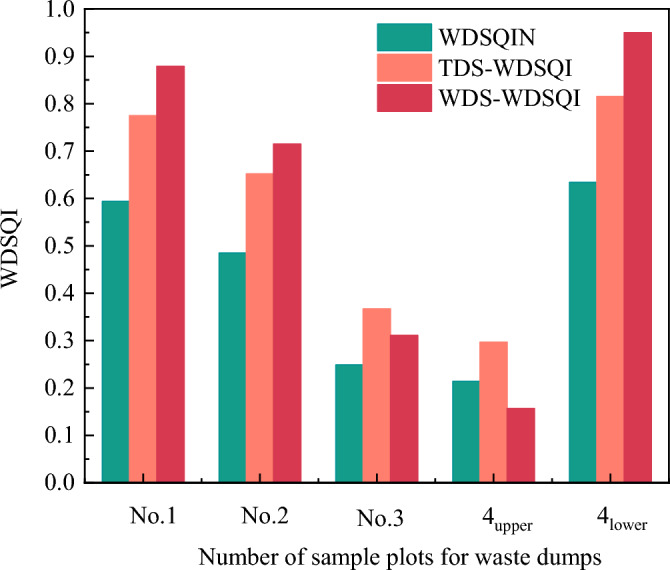
Soil quality index of waste dumps under three evaluation methods.

The improved Nemerow index and membership function were employed to create a comprehensive evaluation model for SQ. The soil fertility was evaluated in five sample plots at four waste dumps. The SQIs for each plot were as follows in Fig. 5.

The WDSQIN of waste dumps in the Hequ open-pit mine is greater for the reclaimed No.1, No.2, and No.4_lower_, whereas the lower values are for No.3 and No.4upper. The Nemerow index ranked the $WDSQIN$ of five sample plots as follows: No.4_lower_ (0.634) > No.1 (0.594) > No.2 (0.485) > No.3 (0.249) > No.4_upper_ (0.214). The No.4_lower_ had a moderate fertility rate. The fertility level of the No.1 and No. 2 had a deficient fertility rate. The No.3 and No.4_upper_ exhibited a fertility quality below 0.4, indicating destitution, with values of 0.249 and 0.214 respectively. The average value stood at 0.4352 ± 0.194. The soil in the waste dumps typically lacking in quality. The $TDS - WDSQI$ of five sample plots was ranked as: No.4_lower_ (0.378) > No.1(0.347) > No.2(0.260) > No.3 (0.174) > No.4_upper_ (0.131). The No.3 and No.4_upper_ exhibited a destitute fertility quality level, falling below 0.4 with 0.367 and 0.297 respectively. The average value stood at 0.258 ± 0.107. In general, the soil in the waste dumps was at a deficient level. The $MDS - WDSQI$ of five sample plots was ranked as follows: No.4_lower_ (0.625) > No.1 (0.518) > No.2 (0.268) > No.3 (0.168) > No.4_upper_ (0.676). The No.3 and No.4_upper_ had a fertility quality level of below 0.4, with 0.311 and 0.157 respectively, indicating that they were in a state of extreme poverty, with an average value of 0.454 ± 0.217. The soil in the waste dumps was generally at a deficient level. The consistency of the three calculation methods was commendable. In essence, the SQ of various waste dumps in Hequ open-pit coal mine waste dumps exhibits a consistent pattern, with No.4_lower_ > No.1 > No.2 > No.3 > No.4_upper_. The data showed that the SQ of the reclaimed area's waste dumps was much higher than that of the unreclaimed area. Specifically, the No.4_lower_ had the most favorable soil conditions. Overall, the waste dumps in the research area exhibited a subpar level of soil quality. The waste dumps of the Hequ open-pit coal mine required immediate soil reclamation and management.

## Discussion

In this study, the approach evaluated the SQ of five sample sites in the Hequ open-pit coal mine waste dumps using two methods: the Weighted Additive Index and the improved Nemorow Index established by experts in the indicators to be included. The WDSQI obtained by the two methods have strong consistency, and both methods are practical and effective.

The TDS consisted of nine soil indicators, specifically SOM, pH, AN, AP, AK, TP, SMC, BD, and TN. Through the integration of PCA and Norm values, the MDS for constructing the soil quality evaluation of the Hequ open-pit coal mine waste dumps was determined based on only three indicators: SOM, pH, and BD. The fitting relationship indicated that the MDS is a viable substitute for the TDS. The MDS was capable of mirroring the assessment data of different metrics in TDS regarding soil quality indicators in waste dumps. This aligns with current studies^14–17^, which obtain the MDS through PCA to obtain the obstacle factors and further investigate the current research status by implementing governance measures for obstacle factors.

Additionally, the approach creatively incorporated the improved Nemerow index method^32,33^ for assessing soil quality. The Nemerow index, as one of the most commonly used methods for calculating comprehensive pollution indicators, is a weighted multi-factor environmental quality index that considers extreme figures or emphasizes the highest values. It particularly considers the factors with the greatest impact and can highlight the role of heavy metal pollution with heavy pollution. According to the "barrel theory", the overall fertility of soil often depends on the nutrient index with the most scarce content. Therefore, the use of the Nemerow index method can clarify the main obstacle indicators to the restoration of soil fertility in the open-pit coal mine dumping site, providing targeted management ideas for soil improvement in the later stage of waste dumps. Based on the nonlinear membership function method and the improved Nemerow index evaluation method, a comprehensive evaluation of soil fertility quality level was conducted for 9 nutrient indicators. The research revealed that the SQ of Hequ's open-pit coal mine waste dumps was primarily influenced by pH levels, AN, AP, SMC, and BD. TP and TN stand as the main limitations to soil fertility SQ in regions not yet unreclaimed.

These findings suggested that these SQIs precisely gauged the effects of reclamation on SQ in terms of both sensitivity and precision. The soil quality index of No.4_lower_ was the highest, indicating that the reclamation significantly increases SQ. Besides, the Nemerow index had a more focused approach and can identify the obstacle factors in each sample area. But overall, the open-pit mine waste dumps had an inadequate SQ. The land that had been reclaimed did not yield the desired results. Essential nutrients continued to be scarce in the Soil. Comparable to soil that remains unreclaimed, there was still a need to fortify it for better governance.

SQI proved to be a valuable instrument for evaluating the effects of soil management practices on SQ. The results of the scoring comparison differed based on the indicator. Both methods provided equal justification for certain indicators. The observed range had a significant impact on the results obtained for the MDS. Overall, the principal components extracted by PCA may make it difficult to explain their meanings, and additional analysis and interpretation are needed to draw conclusions, subject to limitations in sample size and number of variables. If the sample size is insufficient or there are too many variables, it may lead to the extracted principal components not being representative. The improved Nemerow index seems to better represent the functionality of many indicators. And it could better reflect the main obstacles of each sample site It is suggested that an SQIN model be created for the majority of open-pit coal mines, the primary impediments in the research area should be identified, and remedial actions should be taken accordingly.

Our study has several limitations. The limitation of the number of measurement indicators resulted in the omission of broader and more detailed metrics. Future studies aim to broaden the range of indicators, with a particular focus on biological indicators. Microorganisms in the soil persistently break down organic matter and also facilitate the conversion and recycling of nutrients like organic carbon and nitrogen in the soil. These elements primarily contribute to the generation of trace gases in soil and are crucial in managing pollution from organic materials and heavy metals. Consequently, future studies will enhance the assessment index collection, encompassing not just physical and chemical characteristics, but also biological indicators to more accurately represent the soil quality at the waste dumps.

## Conclusion

In response to the actual situation of the Hequ open-pit mine, chemical technologies such as sulfur powder, humic acid fertilizers, and aluminum sulfate can be used to reduce the pH value of the soil in the Hequ open-pit mine waste dumps. Leguminous nitrogen-fixing shrubs such as alfalfa can be planted, as well as non-leguminous nitrogen-fixing shrubs such as sea buckthorn, camphor pine, and alfalfa, in order to improve their overall quality.

## Data Availability

All data generated or analyzed during this study are included in this published article.
